# Grain Size Selection Using Novel Functional Markers Targeting 14 Genes in Rice

**DOI:** 10.1186/s12284-020-00427-y

**Published:** 2020-09-09

**Authors:** Lin Zhang, Bin Ma, Zhong Bian, Xiaoyuan Li, Changquan Zhang, Jiyun Liu, Qun Li, Qiaoquan Liu, Zuhua He

**Affiliations:** 1 Jiangsu Key Laboratory of Crop Genomics and Molecular Breeding /Joint International Research Laboratory of Agriculture and Agri-Product Safety of the Ministry of Education, Yangzhou University, Yangzhou, 225009 China; 2 Jiangsu Co-Innovation Center for Modern Production Technology of Grain Crops, Jiangsu Key Laboratory of Crop Genetics and Physiology, Yangzhou University, Yangzhou, 225009 China; 3 School of Life Science and Technology, Shanghai Tech University, Shanghai, 201210 China; 4 National Key Laboratory of Plant Molecular Genetics, CAS Center for Excellence in Molecular Plant Sciences, Shanghai Institute of Plant Physiology & Ecology, Chinese Academy of Sciences, Shanghai, 200032 China

**Keywords:** Rice, Grain size, Functional nucleotide polymorphism, Functional marker, QTL analysis

## Abstract

**Background:**

Grain size is an extremely important aspect of rice breeding, affecting both grain yield and quality traits. It is controlled by multiple genes and tracking these genes in breeding schemes should expedite selection of lines with superior grain yield and quality, thus it is essential to develop robust, efficient markers.

**Result:**

In this study, 14 genes related to grain size (*GW2*, *GS2*, *qLGY3*, *GS3*, *GL3.1*, *TGW3*, *GS5*, *GW5*, *GS6*, *TGW6*, *GW6a*, *GLW7*, *GL7* and *GW8*) were selected for functional marker development. Twenty-one PCR-gel-based markers were developed to genotype the candidate functional nucleotide polymorphisms (FNPs) of these genes, and all markers can effectively recognize the corresponding allele types. To test the allele effects of different FNPs, a global collection of rice cultivars including 257 accessions from the Rice Diversity Panel 1 was used for allele mining, and four grain-size-related traits were investigated at two planting locations. Three FNPs for *GW2*, *GS2* and *GL3.1* were genotyped as rare alleles only found in cultivars with notably large grains, and the allele contributions of the remaining FNPs were clarified in both the *indica* and *japonica* subspecies. Significant trait contributions were found for most of the FNPs, especially *GS3*, *GW5* and *GL7*. Of note, *GW5* could function as a key regulator to coordinate the performance of other grain size genes. The allele effects of several FNPs were also tested by QTL analysis using an F_2_ population, and *GW5* was further identified as the major locus with the largest contribution to grain width and length to width ratio.

**Conclusions:**

The functional markers are robust for genotyping different cultivars and may facilitate the rational design of grain size to achieve a balance between grain yield and quality in future rice breeding efforts.

## Background

Grain size is the major determinant of grain weight, one of the three yield components (panicle number, grain number and grain weight) in rice, and it can be further categorized into grain length, grain width and length to width ratio (Xing and Zhang 2010). Grain size also correlates with grain quality traits, such as grain appearance, head rice yield, cooking and eating quality (Nelson et al. 2011; Tan et al. 2000). The desirable grain appearance varies in different regions of the world; long and slender rice is preferred by consumers in America, Africa and Southeast Asia, while short and bold rice is preferred by northern China, Japan and Korea (Calingacion et al. 2014). Therefore, it is important to breed rice cultivars with various grain sizes to meet the needs of different market classes.

Grain size is controlled by multiple quantitative trait loci (QTLs), and the chromosomal regions of different loci have been repeatedly detected by genetic populations with different parent combinations (Segami et al. 2016; Xia et al. 2017). Many QTLs for grain size have each been fine-mapped to small chromosomal regions, laying the foundation for gene cloning (Dong et al. 2018; Qiu et al. 2012). In recent years, more than ten QTLs for grain size and weight have been cloned and functionally dissected, including *GW2* (Song et al. 2007), *GS2*/*GL2* (Che et al. 2016; Duan et al. 2016; Hu et al. 2015), *GS3* (Fan et al. 2006; Mao et al. 2010), *qLGY3*/*OsLG3b* (Liu et al. 2018; Yu et al. 2018), *TGW3*/*qTGW3*/*GL3.3* (Hu et al. 2018; Xia et al. 2018; Ying et al. 2018), *GL3.1*/*qGL3* (Qi et al. 2012; Zhang et al. 2012), *GS5* (Li et al. 2011), *GW5*/*qSW5* (Duan et al. 2017; Liu et al. 2017; Shomura et al. 2008; Weng et al. 2008), *TGW6* (Ishimaru et al. 2013), *GW6a* (Song et al. 2015), *GL7*/*GW7* (Wang et al. 2015a; Wang et al. 2015b) and *GW8* (Wang et al. 2012). The QTLs differ in their effects on determining the grain width and grain length, making an improvement either for grain yield or grain quality. *gw2*, *GS5* and *gw5* primarily contributed to increased grain width and grain weight (Li et al. 2011; Song et al. 2007; Weng et al. 2008), while *qlgy3*, *gs3*, *gl3.1* and *tgw3* primarily contributed to increased grain length and grain weight (Fan et al. 2006; Hu et al. 2018; Liu et al. 2018; Qi et al. 2012). *GS2* caused a simultaneous and substantial increase in grain length, grain width and grain weight, but this resulted in poor grain appearance with a significant increase in grain chalkiness (Hu et al. 2015). *GL7* contributed to increased grain length but decreased grain width without affecting grain weight and was designated a grain quality gene for its effect in reducing grain chalkiness (Wang et al. 2015b). *GW8* contributed to an increase in both grain width and weight for yield improvement, and the *gw8* allele reduced grain weight but increased grain length and grain quality (Wang et al. 2012). Genome-wide association studies (GWAS) have been shown to be another efficient method for QTL mapping, and through GWAS, the role played by *GLW7* in determining both rice grain length and weight was first identified (Si et al. 2016). *GS6* was cloned from an EMS mutant, and natural variations of the gene were found to increase grain width and weight (Sun et al. 2013).

Cloning of several different QTLs helped to elucidate the mechanism of grain size determination. Functional analysis demonstrated that grain size traits can be directly linked to the gene coding region or regulatory elements. *GW2*, *qLGY3*, *GS3*, *GL3.1*, *TGW3*, *GW5*, *GS6* and *TGW6* are negative regulators of grain size, while *GS2*, *GS5*, *GW6a*, *GLW7*, *GL7* and *GW8* are positive regulators. A 1-bp deletion in exon 4 of *GW2* resulted in increased grain width, and several SNPs in its promoter also contributed to trait variation (Lu et al. 2013; Song et al. 2007). Similar to *GW2*, one nucleotide deletion was identified in the coding region of *TGW6*, which led to loss of function for the gene and an increase in grain weight (Ishimaru et al. 2013). A T to A nucleotide substitution in exon 2 of *GS3* induced a premature codon, contributing to the increase in grain length (Fan et al. 2006). Additional variations in the coding region of *GS3* have been found in different cultivars, and their effects have not been clarified (Mao et al. 2010). A C to A nucleotide substitution led to an amino acid change in the kelch domain of *GL3.1* and was shown to be the causative polymorphism for long grains (Zhang et al. 2012). The 1212-bp deletion of *GW5* increased grain width directly, because the deletion reduced the expression of its causative gene (Liu et al. 2017; Weng et al. 2008). A truncating splice-site mutation of *qLGY3* was associated with slender grains, and the alternatively spliced protein became a dominant negative regulator of the normal protein (Liu et al. 2018). Functional loss of *TGW3* contributed to an increase in grain length, and several FNPs have been identified from different cultivars (Hu et al. 2018; Xia et al. 2018; Ying et al. 2018). Three haplotypes of *GS6* were identified in natural accessions, and the sequence polymorphism of its promoter most likely determined grain size due to the effect on gene expression (Sun et al. 2013). For the positive regulators, the contributing FNPs were primarily located in the regulatory regions, and polymorphisms in the promoter of *GS5* and *GW6a* and the 5’UTR (untranslated region) of *GLW7* and *GW8* possibly increased grain size by elevating gene expression (Li et al. 2011; Si et al. 2016; Song et al. 2015; Wang et al. 2012). *GS2* is under the control of miR396, and two SNPs have been identified on the miRNA targeting site to generate larger grains by promoting gene expression (Hu et al. 2015). The *GL7* locus harbored a new copy of the original gene, leading to gene upregulation and increased grain length (Wang et al. 2015b). Polymorphisms in the 5’UTR of *GL7* might also determine the trait variation, as they showed distinct ability in driving reporter expression (Wang et al. 2015a).

Clarification of variations controlling grain size traits provides valuable targets for molecular marker-assisted selection (MAS) breeding, and introgression of one or multiple genes has been successful in improving the grain yield and grain quality of different cultivars (Nan et al. 2018; Zhao et al. 2018). Moreover, a rational design breeding strategy was developed and achieved a breakthrough, highlighting the power of pyramiding suitable genes to quickly breed desirable rice cultivars (Zeng et al. 2017). Facilitated by markers linked to genes controlling grain size, eating quality and yield, the Chinese breeders introgressed multiple superior alleles of Nipponbare (NIP) and 9311 into the cultivar Teqing, and obtained improved cultivars with similar yield to Teqing while improving eating quality and appearance (Zeng et al. 2017). During MAS breeding, PCR agarose gel-based markers are popular in most breeding programs because this technology is easy and cost-effective to implement (Hu et al. 2020). Insertion/deletion (InDel) are major sequence variations targeted for marker development, and primers can be easily designed for polymerase chain reaction (PCR). Nevertheless, single nucleotide polymorphisms (SNPs) can also be discriminated by agarose gel using the tetra primer PCR method or cleaved amplified polymorphic sequence (CAPS) markers (Fan et al. 2006; Kim et al. 2016). To date, some gene-linked random markers and functional markers have been developed, involving more than 60 known genes related to yield and biotic and abiotic stress tolerance (Hu et al. 2020; Kim et al. 2016). Compared with gene-linked random markers, functional markers are more efficient in MAS, as they can fix gene alleles in multiple genetic backgrounds without additional calibration (Bagge et al. 2007). Although FNPs of different grain size genes have been identified, very few have been transferred into functional markers (Fan et al. 2006; Kim et al. 2016; Wang et al. 2011; Weng et al. 2008). Therefore, functional makers should be rapidly developed and continuously updated to facilitate breeding applications.

In this study, we surveyed 20 candidate FNPs from 14 cloned grain size genes, and transferred them into PCR-gel based markers. We attempted to clarify the markers’ ability to discriminate the allelic distribution of different FNPs with 257 accessions from the Rice Diversity Panel 1 (RDP1), and evaluated their contribution to modifying grain dimensions (length, width, length to width ratio). Genotyping and phenotyping this large set of accessions made it possible to compare the effect of different FNPs synchronously, thus providing guidance for the rational design of grain size. Moreover, to determine the potential function of the marker set in locating QTLs, we tested it by an F_2_ population. We believe these newly developed functional markers will facilitate both genetic study and breeding applications in the future.

## Result

### Survey of Candidate Functional Variations of 14 Grain Size Genes

**Fig. 1:**
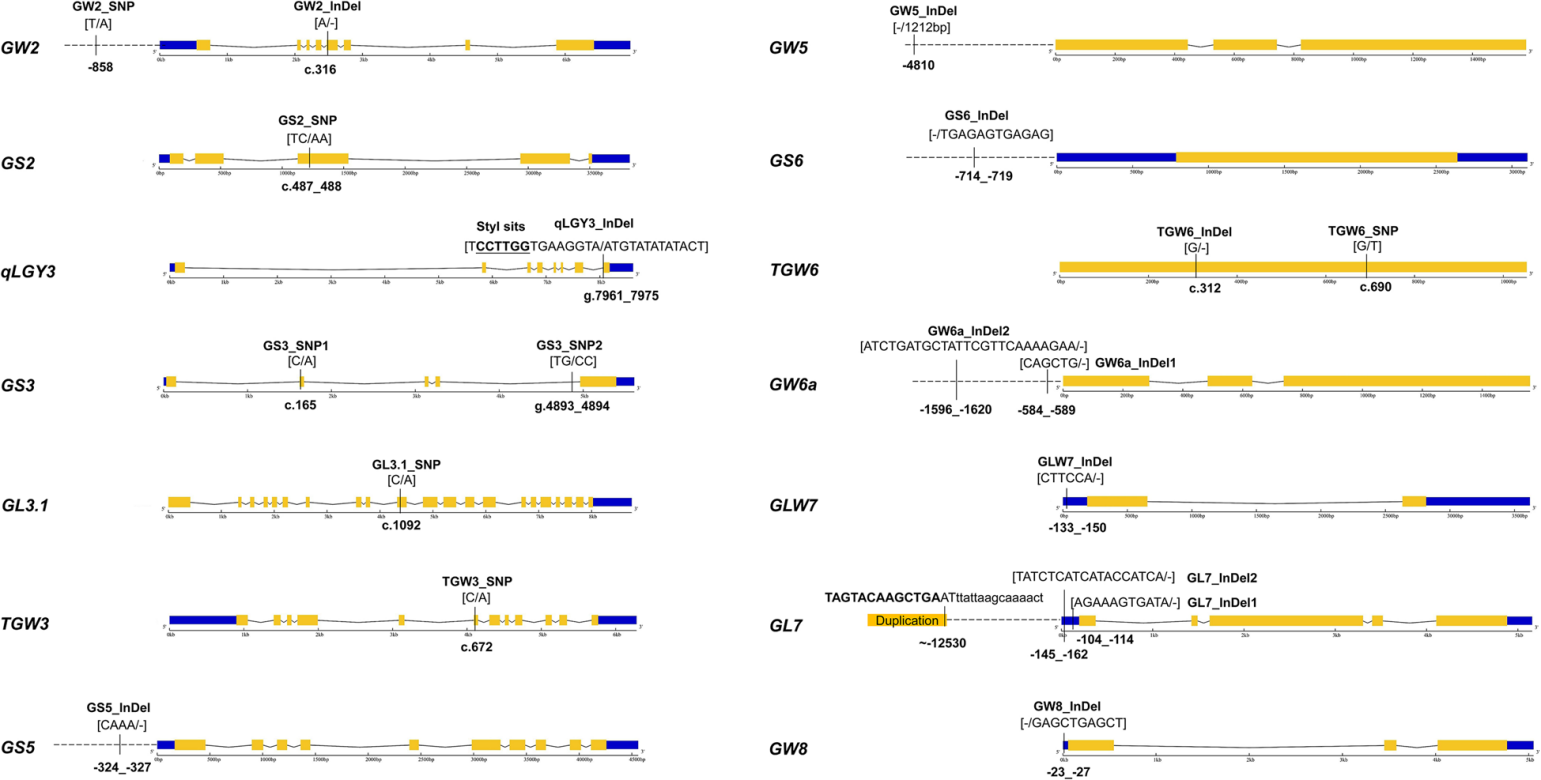
Allele type and position of candidate FNPs of 14 grain size genes. The promoters, UTRs, exons and introns of different genes are expressed as dashed lines, blue boxes, orange boxes and fold lines respectively. The position of each variation is labeled the relative distance to the ATG in promoter (−), CDS (c) and genomic DNA (g). Allele variation of each gene is shown in square bracket, with the front nucleotide variation representing allele 1 from Nipponbare and the back nucleotide variation representing allele 2 from non-NIP-type cultivars

According to previous reports, 14 grain size QTLs/genes located on six chromosomes were selected for sequence verification (Fig. 1 and Table S1). Using the Nipponbare (NIP) genome as the reference, we confirmed the polymorphic sequences and their positions on different genes, and 20 candidate FNPs including seven SNPs and 13 InDels were selected (Fig. 1). The contributions of several FNPs are known for respective genes, including the 1-bp deletion in exon 5 of *GW2*, the TC to AA substitution in exon 3 of *GS2*, the replacement of 15-bp sequence by 14-bp sequence in the joint region of intron 7 and exon 8 of *qLGY3*, the C to A substitution in exon 2 of *GS3*, the C to A substitution in exon 10 of *GL3.1*, the C to A substitution in exon 6 of *TGW3*, the 1212-bp deletion in promoter of *GW5*, the 1-bp deletion in the single exon of *TGW6*, the 6-bp/10-bp InDels in the 5’UTR of *GLW7*/*GW8* and the *GL7* duplication approximately 12.5 kb upstream of its original copy (Fig. 1). The 4-bp InDel of the *GS5* promoter had been designated a functional variation without further validation (Kim et al. 2016) and was subjected to marker development to validate its effect. A T to A SNP at the − 858-bp position of the *GW2* promoter was selected as a candidate functional variation, which was identified in a previously reported haplotype analysis (Lu et al. 2013). A 3-bp deletion with an unknown effect existed in exon 5 of *GS3* in the cultivar Zhenshan 97 (Mao et al. 2010), and we sequenced 43 RDP1 accessions to check its allele frequency. Ten accessions harbored the deletion tightly linked with the 2-bp SNP substitution in intron 4, which was used for marker design (Fig. 1 and Fig. S1). In addition to the 1-bp deletion of *TGW6*, six SNPs were also identified from the coding region between Kasalath and NIP (Ishimaru et al. 2013), and the G to T SNP at the 690-bp position of the exon was selected for marker design to test its effect (Fig. 1). Sequence polymorphisms from the − 395 to − 1415 position of the *GW6a* promoter have been suggested to contribute to trait variation (Song et al. 2015), and a 6-bp deletion in that interval and another 25-bp deletion located at approximately the − 1.6 kb position were selected for marker design to compare their effects (Fig. 1). One 12-bp insertion in the *GS6* promoter and two deletions in the 5’UTR of *GL7* likely affect the grain size by changing their gene expression (Sun et al. 2013; Wang et al. 2015a), and were selected for marker development to validate their effect (Fig. 1). To facilitate citation, all the sequence variations were named the gene name with the mutant type (Fig. 1), allele 1 represented the reference allele based on the NIP sequence, and allele 2 represented another allele type (i.e. non-NIP-type illustrated in Fig. 1 legend).

### Marker Development and Quality Evaluation for Gel Electrophoresis

To facilitate large-scale genotyping, we attempted to transfer all the candidate FNPs into PCR-gel-based molecular markers. InDels larger than 10 bp can be identified by PCR directly using flanking primers on the condition that the size of PCR products is no more than tenfold greater than the InDel size (Yamaki et al. 2013). With this criteria, six InDel markers for GW5_InDel, GS6_InDel, GW6a_InDel2, GL7_InDel1, GL7_InDel2 and GW8_InDel were developed (Table 1). In addition to the 6-bp deletion of the *GLW7* 5’UTR, we found an additional 3-bp deletion in some accessions (Fig. S2), and based on this, an InDel marker covering the two small InDels was developed. We also developed new primers flanking the border of the *GL7* duplication with PCR products smaller than those previously reported (Wang et al. 2015b), which can save PCR amplification time. CAPS or dCAPS markers were developed for the remaining variations including the 6-bp deletion of *GLW7*. In the target region of *qLGY3*, the reference allele harbors a StyI enzyme site, which is aborted in the mutated sequence (Fig. 1), and the CAPS marker was generated by a pair of flanking primers (Table 1). For dCAPS markers, one or two nucleotides in the forward primers were replaced by other types to generate a restriction enzyme site, and the strategy was successful for both SNPs and small InDels (Fig. S3). Finally, twelve forward dCAPS primers involving 10 restriction enzyme sites were selected (Fig. S3), and the reverse primers were designed accordingly. Taken together, twenty-one pairs of primers were developed for the 20 FNPs (Table 1).

**Table 1:** Primer information on the 21 functional markers targeting 14 grain size genes

Primer Names	Primers sequence (5′-3′)	Enzyme sites	Amplicon size (Digested) bp
GW2_SNP-F	AAAACCAAAACCTAACACGTGGATACAACA	NspI	115 (84)
GW2_SNP-R	GGCGGTGAAGATAGATGTACT		
GW2_InDel-F	CTCACACTGCTCAGCCTACA	PstI	158 (127)
GW2_InDel-R	CACGATACTCCACAGCATAACTGGGAGTCT		
GS2_SNP-F	GAGCGCCACATGCACCGCGGCCGCATACGT	SnaBI	287 (255)
GS2_SNP-R	TTGCCTGTTCCACCACCAACAGC		
qLGY3_InDel-F	GGGTATTCTTCTTTTCTTCCA	StyI	448 (371)
qLGY3_InDel-R	TGTCTGCTGCTTCATTGCT		
GS3_SNP1-F	CGAAGGGATCCACGCTGCCTCCAGATGATT	HinfI	170 (140)
GS3_SNP1-R	AGTTGCTTAAAAAGATAACGGTCAAA		
GS3_SNP2-F	CAAACATGAAAACCTTGTCT	HaeIII	186 (155)
GS3_SNP2-R	AAATAAAACGTGTGATTTAATCGTTAACGGC		
GL3.1_SNP-F	TGCACGATTCTATCTGGTTCAGTGGTCGA	SalI	224 (193)
GL3.1_SNP-R	CTAAACAAACAGGTTTTCTTAC		
TGW3_SNP-F	TGCACTGCCAAAATCACATATTTTGAGCCG	HpaII	299 (269)
TGW3_SNP-R	TCAACTACCACTAGGTCCAC		
GS5_InDel-F	ACTCCCATGGAATTACTAGAGAAGCCAAGC	AluI	262 (205/172)
GS5_InDel-R	AGGAGAAGAAAAGGTGAAAAGT		
GW5_InDel-F	TGCGTCGGTCGTTGGAGG		419/1631
GW5_InDel-R	GCGAGCGAGCGTGTGTAGG		
GS6_InDel-F	TCTTCTTCCCTTCCCTTCCAC		113/125
GS6_InDel-R	TGCCTCACCATTACCACTCCT		
TGW6_InDel-F	AGCCCCAGCTACACGAAAAACAAGTGCCCG	HpaII	154 (104/72)
TGW6_InDel-R	GACCAACTCGCATCAATCCC		
TGW6_SNP-F	ATGCGAGTTGGTCCAAAAGG	SacI	296 (266)
TGW6_SNP-R	GCAACGATCAGATGTGTTCGGTCAGAGCT		
GW6a_InDel1-F	TGCACCTCTCTCGTGTTTCT	BclI	147 (117)
GW6a_InDel1-R	TTATTTTCGTTCAATTTTTCTATGGCTGAT		
GW6a_InDel2-F	ATTACTCTTGATTTTTGTCTGTT		186/161
GW6a_InDel2-R	GAGTTTGCATGTTCGTTCT		
GLW7_InDel-F	CTCGAGCTCGAGCTCATG		107/101/98
GLW7_InDel-R	TCCCTTTCAACCTTTTCCA		
GLW7_InDel_dCaps-F	CCTCCTCCTCCTCCGCCTTCCACTTCGACT	HinfI	133 (101)
GLW7_InDel_dCaps-R	TGCTACTGTGTGCTGTGTGCT		
GL7_Dup-F	GATGAGCTGACAAGAGCAAAG		0/314
GL7_Dup-R	AGAGGATGGGGATGAAAGAAT		
GL7_InDel1-F	CAGCTCACGCACATCCAAC		112/101
GL7_InDel1-R	ACCATACCACATCTCATCTCAC		
GL7_InDel2-F	GGACGCGTGAGATGAGATGTG		118/100
GL7_InDel2-R	CTCCCGCTTATTTCAACCCC		
GW8_InDel-F	GTGCGTGCGTCAACACACAG		88/98
GW8_InDel-R	TGAGATCCCACTCCATGGCC		

**Fig. 2:**
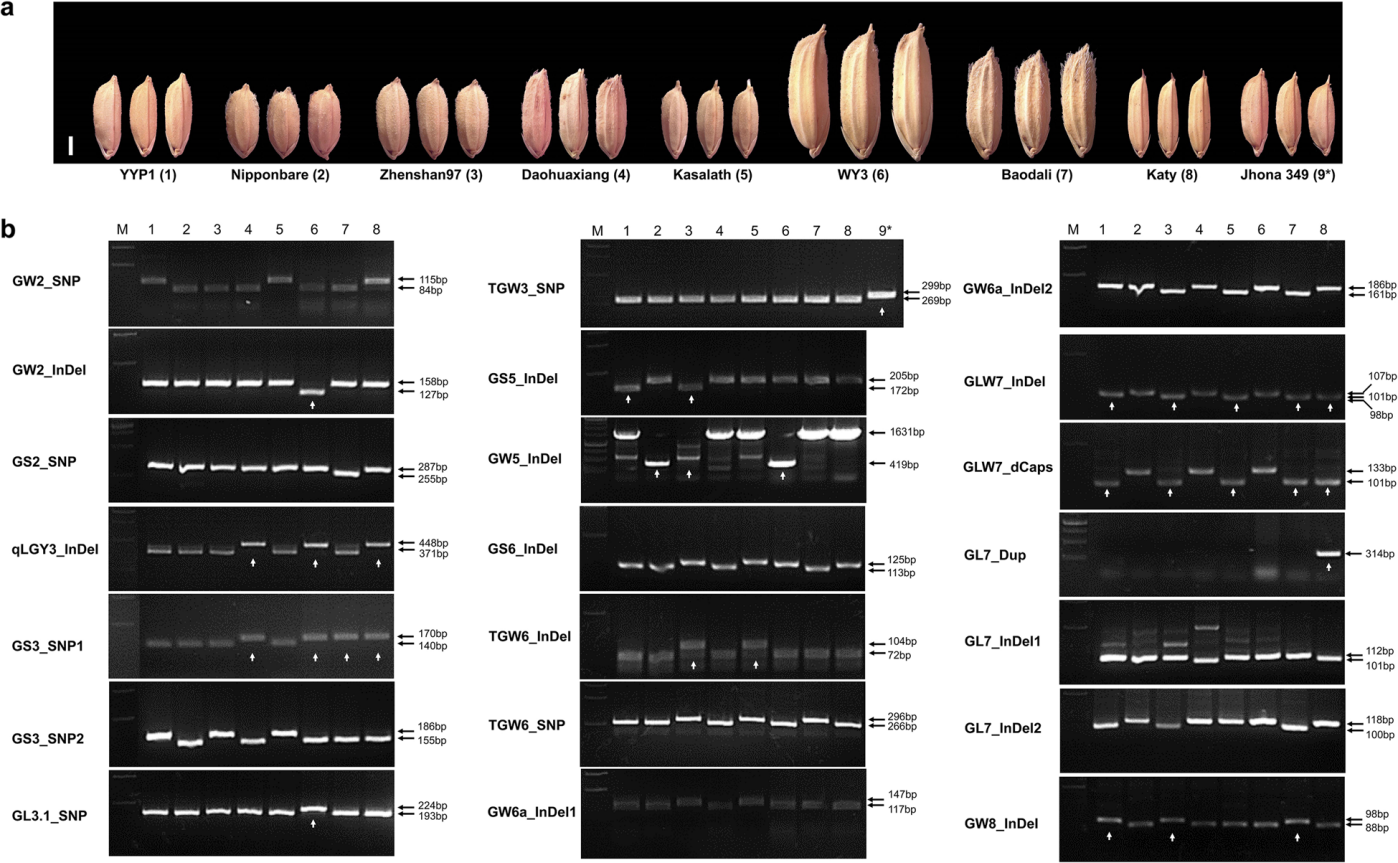
Evaluation of the marker set by PCR-gel-based analysis. a. View of grain size of nine cultivars for marker evaluation. Scale bar, 2 mm. * denotes the cultivar only for allele discrimination of the TGW3_SNP marker. b. Agarose gel images of 21 markers. The band size in bp of the different alleles is labeled on the right side (black arrow), and the superior alleles for wider or longer grains are identified by white arrows. (M identifies the DNA ladder)

To check the markers’ ability to discriminate different alleles, eight cultivars were genotyped with the markers. As no allele variation was found for TGW3_SNP among the eight cultivars, thus an additional cultivar with the target allele was added for the marker (Fig. 2). It is worth noting that WY3 and Baodali are two cultivars with notably large grains (Fig. 2a), which were the original cultivars for cloning *GL3.1*/*GW2* and *GS2*, respectively. All the markers showed good amplicon quality and polymorphism among the tested cultivars (Fig. 2b). For the GL7_Dup marker, a 303-bp band can only be amplified in the U.S. long grain cultivar Katy which has a slender grain, indicating its specificity in amplifying the border region of the new copy. The variation of large InDels can be reflected directly by the amplicon size of InDel markers (Fig. 2b). In the NIP cultivar, smaller bands were amplified for GW5_InDel, GS6_InDel and GW8_InDel, and larger bands were amplified for GW6a_InDel2, GLW7_InDel, GL7_InDel1 and Gl7_InDel2, closely matching the respective InDel size (Fig. 1). As Zhenshan97 harbors another type of *GW5* mutation (Duan et al. 2017), it was identified as a null band by the GW5_Indel marker (Fig. 2b). For the CAPS marker of *qLGY3*, smaller bands were found in the cultivars with the NIP-type allele after enzyme digestion, thereby confirming the existence of the StyI site on the target region (Fig. 1 and Fig. 2b). Eight dCAPS markers showed digestible bands for NIP, including GW2_SNP, GS3_SNP1, GS3_SNP2, GL3.1_SNP, TGW3_SNP, TGW6_InDel, TGW6_SNP, and GW6_InDel2 (Fig. 2b). In contrast, resistant bands for NIP were generated by GW2_InDel, GS2_SNP, GS5_InDel and GLW7_InDel (dCAPS) markers (Fig. 2b). Compared with the InDel marker of *GLW7*, its dCAPS marker was better at discriminating the allele difference (Fig. 2b). Therefore, all these newly developed markers correctly identify allele variations of different grain size genes.

To facilitate comparisons between the markers, we defined the allele for wider or longer grains as the “superior allele”, although the contrasting allele would also be useful depending on the grain shape preference for a particular market. With this in mind, we compared the number of known superior alleles among the tested cultivars and found that each cultivar contained no more than five superior alleles (Fig. 2b; white arrows). Two cultivars with large grains harbored five and four superior alleles, specifically *gw2*, *qlgy3*, *gs3*, *gl3.1*, *gw5* for WY3 and *GS2*, *gs3*, *GLW7* and *GW8* for Baodali (Fig. 2b). Although Zhenshan97 also contained five superior alleles, including *GS5*, *gw5*, *tgw6*, *GLW7* and *GW8* (Fig. 2b), its grain size was significantly smaller than that of WY3 and Baodali (Fig. 2a). It can be inferred that other unknown genes contributed to the increase in grain size for these two large grain cultivars.

### Grain Size Performance of a Large Rice Diversity Panel

**Fig. 3:**
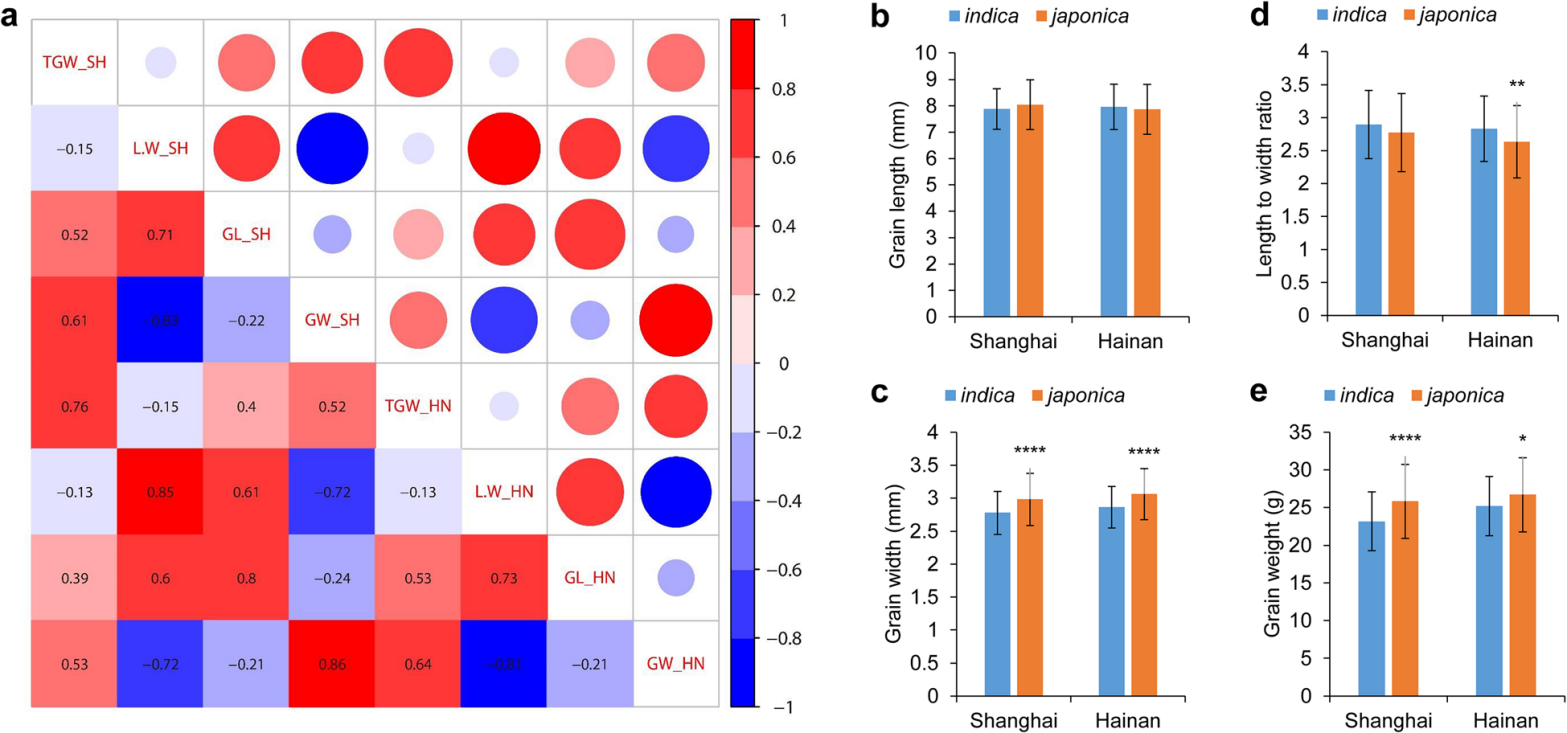
Trait analysis of the RDP1 diversity panel. **a** Correlation analysis of four grain size traits at two locations, Shanghai and Hainan, China. The correlation coefficients are illustrated both by the circle size and color gradient ranging from 1 to −1. Red and blue colors denote positive and negative correlations, respectively, and darker colors and larger circles correspond to greater correlations between traits. **b**-**e** Grain trait measurements between *indica* and *japonica* subspecies at two locations. *, ** and **** indicate significance at *P* < 0.05, *P* < 0.01 and *P* < 0.001 by Student’s t test, respectively

To clarify the allele contribution of different FNPs in shaping grain size, we analyzed 257 accessions in the RDP1 from different regions of the world (Table S2). The diversity panel was planted at Shanghai and Hainan, two locations corresponding to the temperate and tropical climates respectively, which should facilitate the comparison of environmental effects. Mature grains were harvested for trait analyses, including grain length, grain width, grain length to width ratio and grain weight. Pearson correlation analysis was performed for the different traits, and significant correlations were found for the same traits between the two locations, suggesting consistent performance of most accessions in different environments (Fig. 3a). Among the four traits, grain length showed the strongest positive correlation to length to width ratio and a weak negative correlation to grain width, while grain width showed the most significant negative correlation to length to width ratio. Grain weight showed a similar positive correlation with grain length and grain width, and a weak negative correlation with length to width ratio (Fig. 3a). This result suggests that the length to width ratio can be modified easily by changing either grain length or grain width, but it may be difficult to increase the grain weight by improving grain length and width simultaneously.

Based on the previously reported population structure analysis (Table S2; McCouch et al. 2016), we divided RDP1 accessions into the two well recognized rice subspecies, *indica* and *japonica* and compared the trait variations between the subspecies at Shanghai and Hainan. No significant difference in grain length was found between the two subspecies, but a considerable increase in grain width was identified in the *japonica* subspecies at both locations (Fig. 3b, c). For the length to width ratio, a significant decrease in *japonica* subspecies was only found at the Hainan (Fig. 3d). Similar to grain width, the grain weight was increased in the *japonica* subspecies at both locations (Fig. 3e), suggesting that the higher grain weight of these accessions was largely attributable to the grain width.

### Clarification of Allele Effects by Different FNPs

**Fig. 4:**
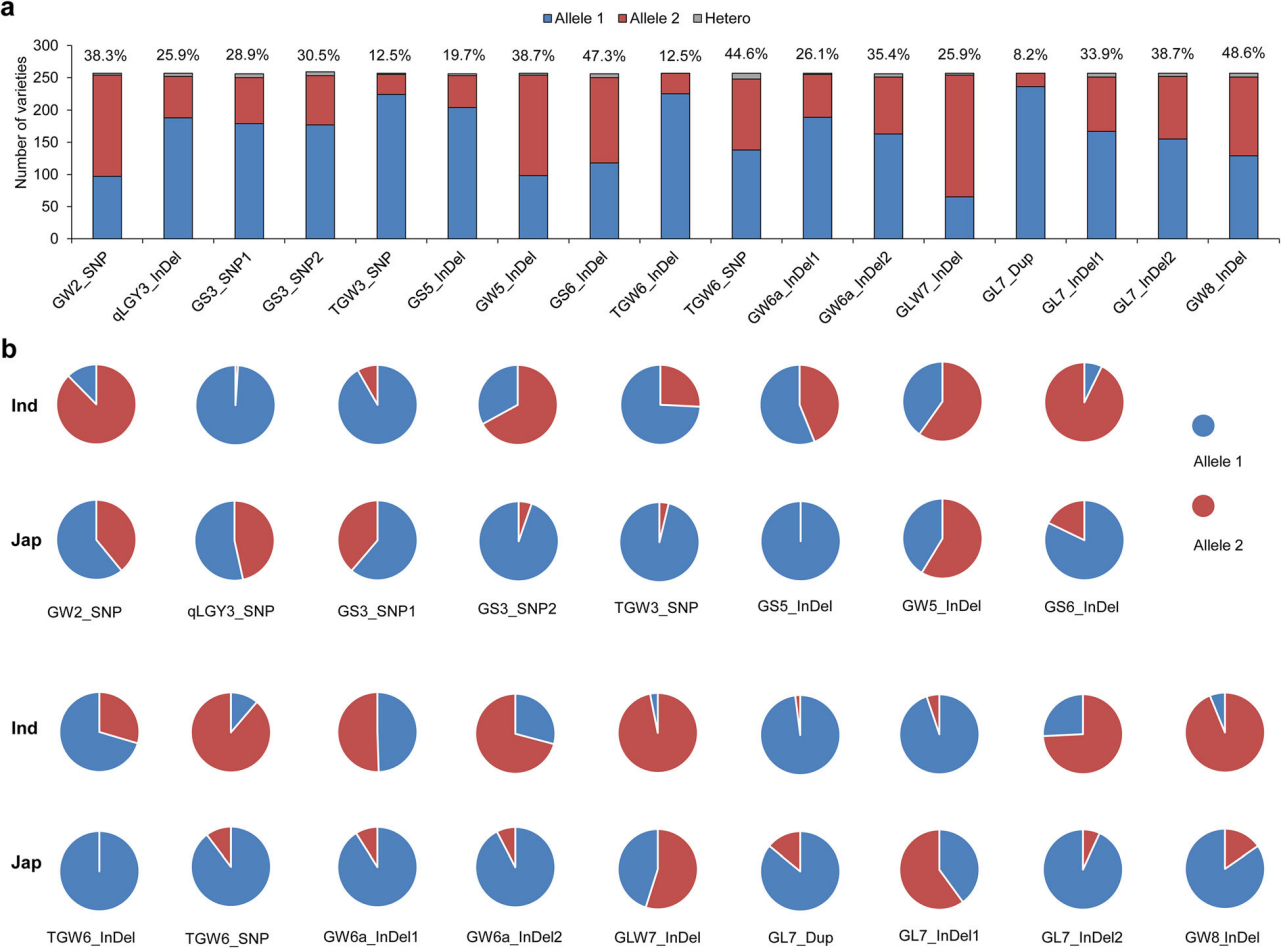
Allele distribution of 17 FNPs in the RDP1 diversity panel. **a** Number of accessions with allele 1 or allele 2 of each FNP in the population. The minor allele frequency is labeled as percent on the top of columns. Allele 1 and Allele 2 represent the sequence variations of NIP- and non-NIP-type accessions respectively, and Hetero represents the heterozygote. **b** Allele ratio of different FNPs in two subspecies by pie chart. Ind and Jap represent *indica* and *japonica* subspecies respectively. Two colors denote the percent of two alleles for each FNP

Using the newly-developed functional markers, we obtained all the allele types of the 20 FNPs for the RDP1 accessions (Table S4). We did not find the superior alleles of GS2_SNP, GW2_InDel and GL3.1_SNP identified from the large grain cultivars, indicating that they belonged to rare alleles. The other polymorphic sites showed relatively balanced allele distribution, with the minor allele frequencies ranging from 8.2% to 48.6%, and GL7_Dup exhibiting the fewest superior alleles (Fig. 4a). In addition, heterozygotes were found in a few accessions (Fig. 4a). We further compared the homozygous allele distribution between the two subspecies and found an uneven allele ratio in either subspecies for most target variations (Fig. 4b). Most *indica* accessions carried allele 1 of qLGY3_SNP, GS3_SNP1, GL7_Dup and GL7_InDel1 variations, and allele 2 of GS6_InDel, TGW6_SNP, GLW7_InDel and GW8_InDel variations. Most *japonica* accessions contain allele 1 of GS3_SNP2, TGW3_SNP, GS5_InDel, TGW6_InDel, TGW6_SNP, GW6a_InDel1, GW6a_InDel2, and GL7_InDel2. Therefore, some useful alleles were enriched only in a specific subspecies.

**Fig. 5:**
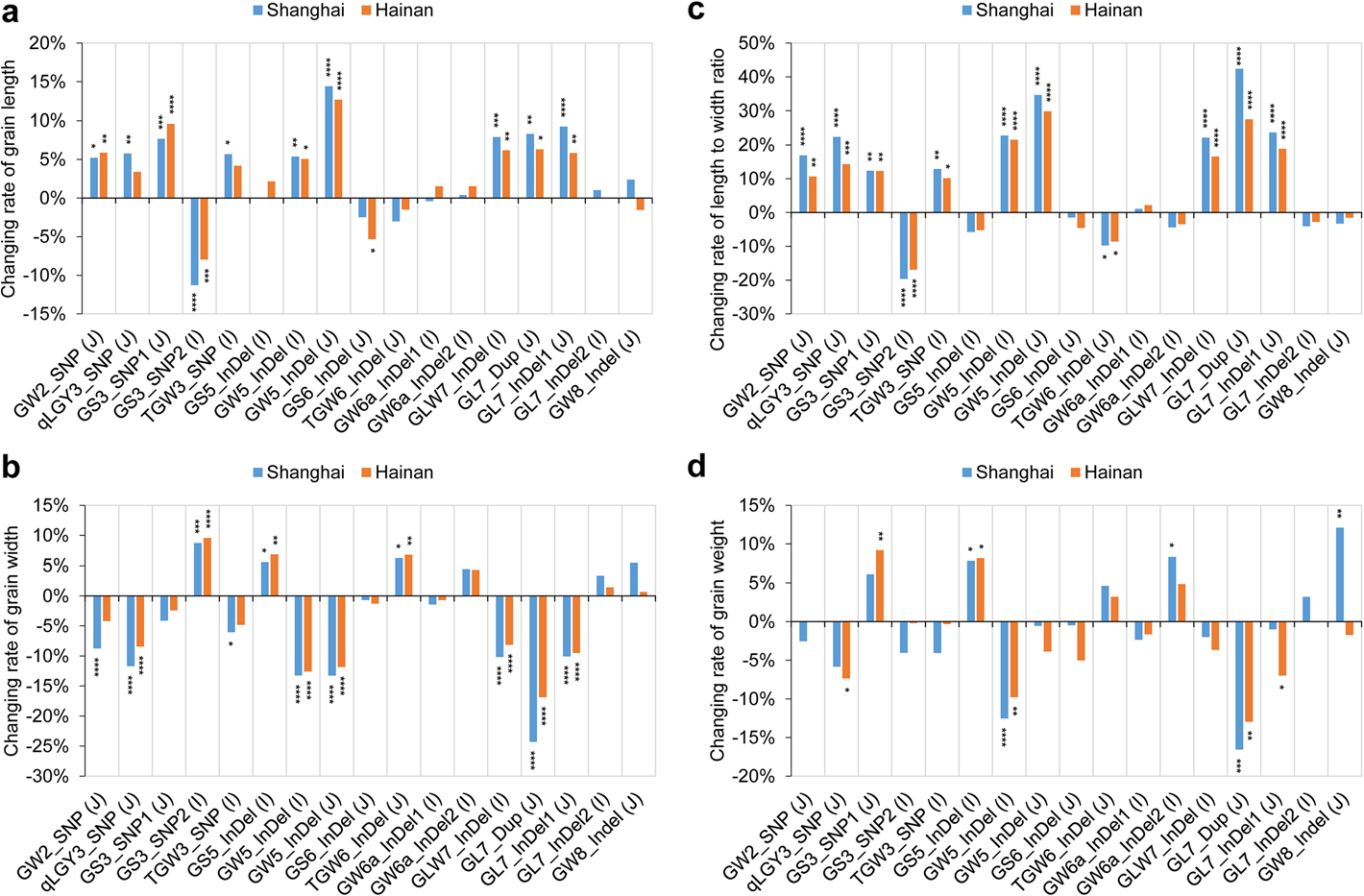
Contribution of different alleles to four grain-size-related traits. The columns show the relative changing rate of allele 2 (non-NIP-type) exceeding allele 1 (NIP-type) for grain length (**a**), grain width (**b**), length to width ratio (**c**) and grain weight (**d**). The I and J in parentheses represent the *indica* and *japonica* subspecies respectively. The values are shown as percentages, and the significance level of the difference between the two alleles of each SNP was calculated by Student’s t test. *, **, *** and **** indicate significance at *P* < 0.05, *P* < 0.01, *P* < 0.001 and *P* < 0.001, respectively

We then compared the contribution of different FNPs in determining grain size, and 16 FNPs were subjected to analysis in either subspecies with a relatively even allele distribution (Fig. 4b). To show the allele contribution directly, we determined the changing rate of mean values of different traits between two alleles, using allele 1 as the reference (Fig. 5). We found that nine FNPs contributed largely to grain length, grain width or length to width ratio, namely GW2_SNP, qLGY3_SNP, GS3_SNP1, GS3_SNP2, TGW3_SNP, GW5_InDel, GLW7_InDel, GL7_Dup and GL7_InDel1 (Fig. 5a-c). Among these FNPs, allele 1 of GS3_SNP2 and allele 2 of the remaining FNPs increased both the grain length and length to width ratio but decreased the grain width. GS5_InDel contributed only to the change in grain width, with allele 2 for the wider grains. GS6_InDel contributed to the change in grain length only at Hainan, with allele 1 for the longer grains. TGW6_InDel contributed to both grain width and length to width ratio, with allele 2 increasing grain width but reducing the length to width ratio. No significant contribution to the three abovementioned traits can be found for GW6a_InDel1, GW6a_InDel2, GL7_InDel2 and GW8_InDel, suggesting relatively weak effects of these variations. Regarding grain weight, stable trait contributions can only be found for GS5_InDel and GW5_InDel in the *indica* subspecies and GL7_Dup in the *japonica* subspecies, with allele 2 of GS5_InDel and allele 1 of GW5_InDel and GL7_Dup increasing the trait (Fig. 5d). Five variations contributed to the trait change only at one location, namely, an increase in grain weight by allele 1 of qLGY3_SNP and GL7_InDel1 and allele 2 of GS3_SNP1 in Hainan, and allele 2 of GW6a_InDel2 and GW8_InDel in Shanghai (Fig. 5d). Therefore, grain weight determination could be complicated due to changes in multiple grain dimensions and easily affected by the environment. Moreover, the effect of GW5_InDel on grain weight can only be found in *indica* subspecies, suggesting that genetic background could affect the performance of different genes.

**Fig. 6:**
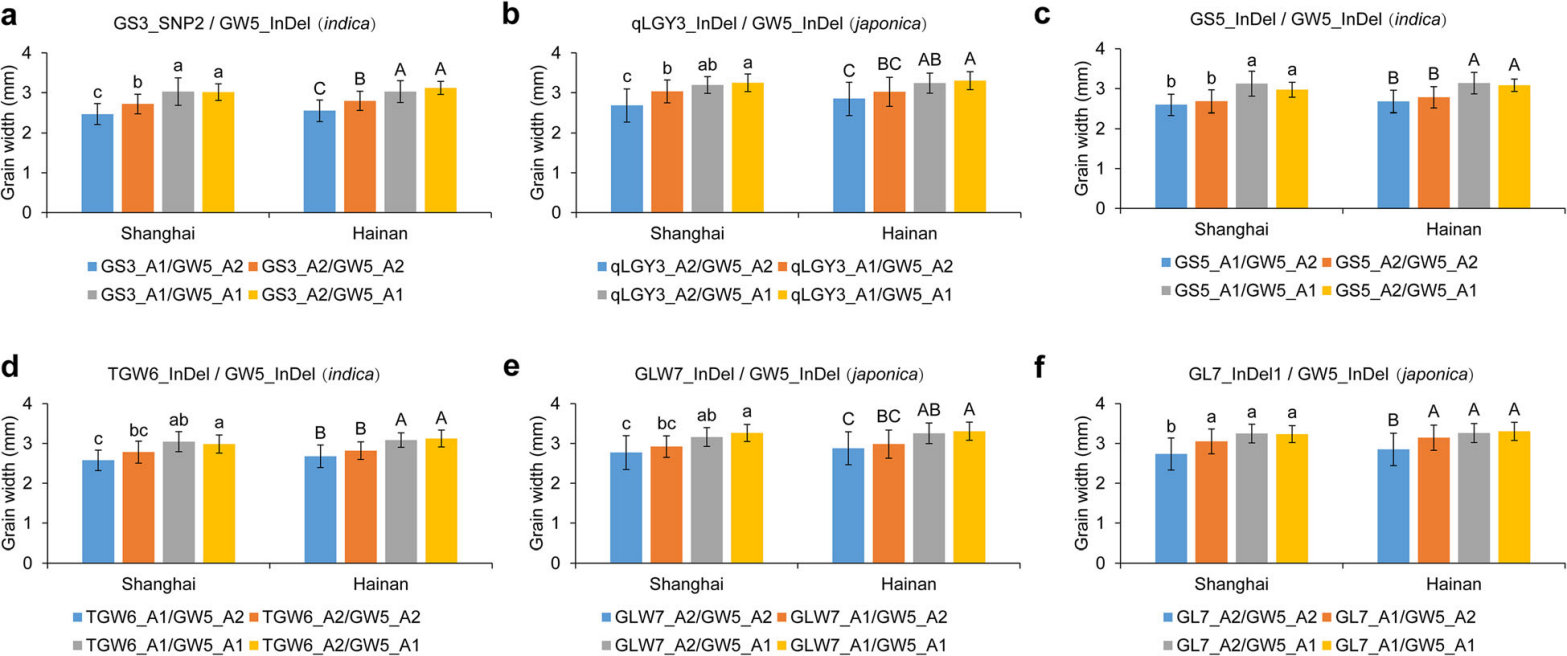
Impact of *GW5* alleles on grain width control of six other loci . Four allele combinations can be obtained between GW5_InDel and six other FNPs in either the *indica* or *japonica* subspecies, namely GS3_SNP2 (**a**), qLGY3_InDel (**b**), GS5_InDel (**c**), TGW6_InDel (**d**), GLW7_InDel (**e**) and GL7_InDel1 (**f**). A1 represents allele 1 (NIP-type) and A2 represents allele 2 (non-NIP-type). Multiple comparisons by Tukey’s test were performed for data from Shanghai and Hainan, and different letters indicate significant differences among the four allele combinations

Among the variations detected, GW5_InDel showed balanced allele distribution and stable effect in determining grain width for both subspecies (Fig. 4b and Fig. 5b), therefore we tested its allelic interactions with other variations, including GS3_SNP2, qLGY3_InDel, GS5_InDel, TGW6_InDel, GLW7_InDel and GL7_InDel1 (Fig. 6). Allele 1 of GW5_InDel had a stable effect by increasing grain width when combined with either alleles of other variations. However, the promoting effect of other tested variations can only be identified when combined with allele 2 of GW5_InDel, especially for GS3_SNP2, qLGY3_InDel and GL7_InDel1. Therefore, allele 2 of *GW5*, namely the functional allele with the 1212-bp sequence, could underlie the regulation of grain size for other QTLs.

### Evaluation of the Functional Markers by QTL Analysis

**Fig. 7:**
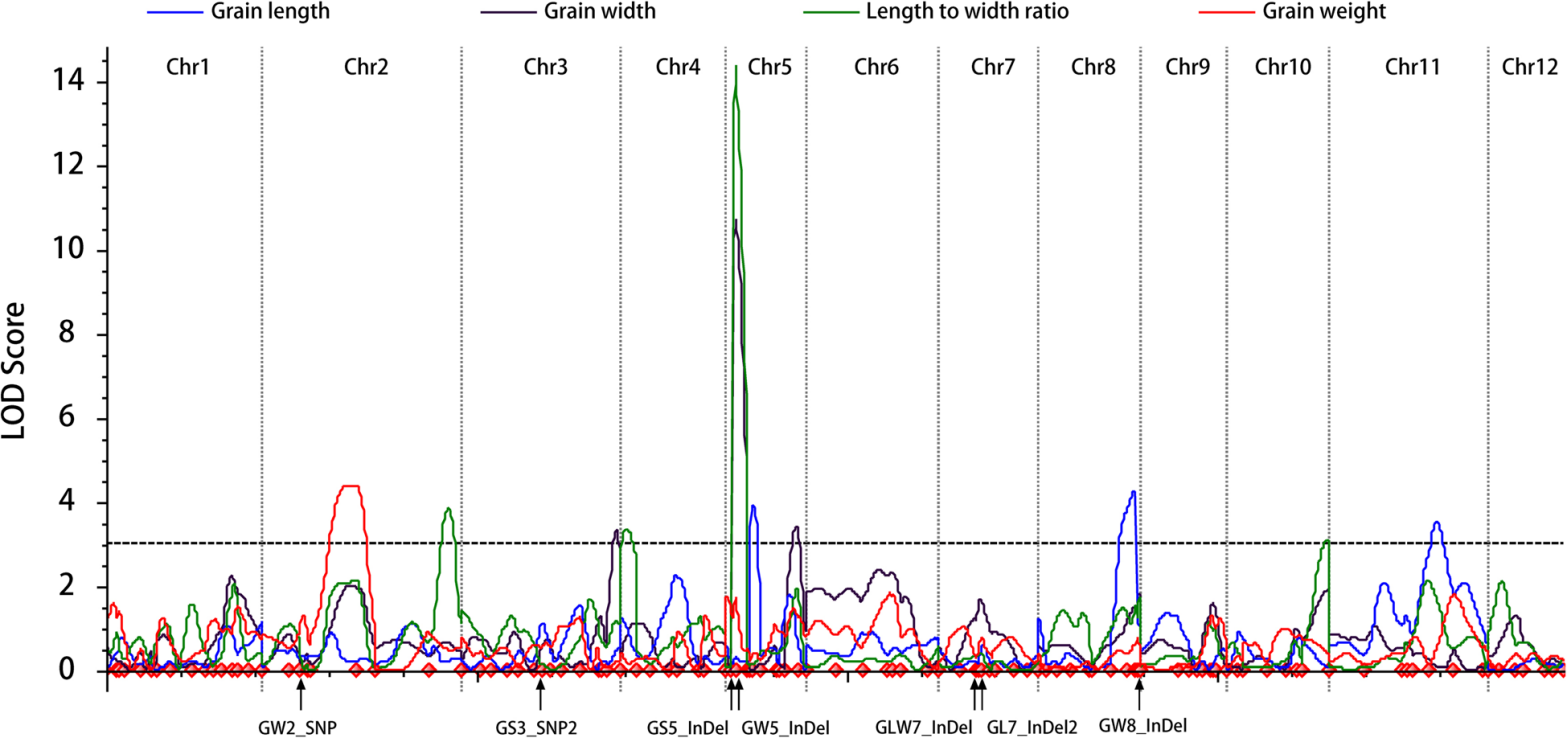
Contribution of polymorphic markers in the QTL analysis. QTL analysis was performed using 123 markers across all 12 chromosomes, and LOD score of the four grain size traits are shown by different colors. The horizontal dashed line indicates the threshold for QTL detection, and peaks over the threshold represent the existence of QTLs. The red diamonds correspond to the position of all the markers used for QTL analysis, and arrows denote positions of seven polymorphic functional markers for grain size

We then tested the allele contribution using an available F_2_ population with 119 individuals derived from a cross of YYP1 and NIP. We found that the two parents had different allele types for GW2_SNP, GS3_SNP2, GS5_InDel, GW5_InDel, GLW7_InDel, GL7_InDel2 and GW8_InDel, and NIP carried allele 1 of GW5_InDel for wider grains (Fig. 2). QTL analysis for the four grain size traits was performed with 123 markers evenly distributed across the 12 chromosomes, which covered the seven polymorphic functional markers (Table S5). In all, 11 QTLs were identified for the four traits, including three for grain length on chromosomes 5, 8 and 11, three for grain width on chromosomes 3 and 5, four for length to width ratio on chromosomes 2, 4, 5 and 10, and one for grain weight on chromosome 2 (Fig. 7 and Table S6). The QTL on the short arm of chromosome 5 presented the largest LOD value for length to width ratio and grain width, and explained ~ 24% and 22% of the phenotypic variation respectively (Table S6). It can be inferred that *GW5* underlies the major QTL, as the GW5_InDel marker colocalizes with the QTL region and the promoting effect is contributed by the NIP allele. No exact QTL colocalization was found for the other functional markers, possibly due to the weak effect of these variations or their epistatic interaction with *GW5* as suggested above. Therefore, *GW5* could be the key locus for controlling grain size and had a stable contribution to grain width and length to width ratio in nearly all genetic backgrounds.

## Discussion

Gene-associated markers are important when implementing the MAS strategy in the breeding process. This was illustrated in rice where gene-linked markers were developed for single or multiple genes for various traits and these were used to develop improved cultivars with purified genetic backgrounds in approximately six rounds of backcrossing without field trait selection (Liu et al. 2016; Wang et al. 2017). Many MAS breeding practices have been performed to target disease or pest resistance genes, and restorer or male sterility lines with improved resistance have been generated (Jiang et al. 2019; Jiang et al. 2015; Wang et al. 2016). However, limited research has been focused on the MAS of yield-related traits in recent decades. Recently, beneficial alleles of the *GS3*, *Gn1a*, and *Hd1* genes were introgressed into Kongyu131, a high-quality cultivar widely planted in the northeastern region of China, which improved both the grain yield and regional adaptation of the newly developed lines (Feng et al. 2017; Nan et al. 2018; Wang et al. 2019). To date, many gene-linked markers have been developed to facilitate MAS breeding but these gene-linked markers are not associated with the target alleles for the genes and can only be used for allele mining after their association with the target allele is confirmed (Hu et al. 2020). In contrast, functional markers developed from the causative variations are diagnostic of the desired trait allele thus are the preferred over gene-linked markers (Liu et al. 2012b). Although several functional markers have been developed, the functional marker set needs to be broadened as additional significant genes are characterized. A functional marker set was developed for eight yield-enhancing genes, but only covered two genes for grain size (Kim et al. 2016). In order to implement more extensive use of MAS breeding, we developed a more extensive functional marker set covering the main cloned QTLs for grain size and proved the effects of these markers in discriminating grain size traits.

All the markers we developed are PCR-gel-based and can be easily implemented by most breeding programs with reasonable equipment expenditures. Among the different DNA polymorphisms, InDels can be easily transferred into molecular markers, but only work efficiently when the InDel size is large enough. As indicated by the GLW7_InDel marker in our analysis, the smallest InDel size could be 9 bp for agarose gel discrimination on the condition that the PCR product is also small. However, four FNPs for the grain size genes have InDels smaller than 9 bp, making them difficult to discriminate as InDel markers. As shown by Kim et al. (2016), the InDel marker for the 4-bp deletion of *GS5* can only be discriminated by polyacrylamide gel electrophoresis or capillary electrophoresis, thus more efficient markers for these small InDels need to be developed by a new strategy. For SNP variations, PCR-gel-based markers can be developed by either the allele-specific PCR method or the CAPS/dCAPS method (Drenkard et al. 2000), but the PCR method has the limitations of nonspecific amplification, being efficient for specific SNP types and a low overall success rate in generating primers (Drenkard et al. 2000; Liu et al. 2012a). Moreover, this method requires two runs of PCR, thus increasing the cost for gel electrophoresis and data collection. Therefore, we used the CAPS/dCAPS method for marker development and successfully transferred all the SNPs into dCAPS markers. Noteworthy, small InDels were also transferred into dCAPS markers, providing a novel strategy for marker design of such FNPs. Utilizing this strategy, it was possible to transfer nearly all the FNPs for different genes into PCR-gel-based markers, thus facilitating breeding efforts to utilize a large number of genes in MAS strategies. The cost of each marker was also considered and priority was given to inexpensive restriction enzymes during primer design. Compared with the previous functional markers, the newly-developed marker set may reduce either the economic cost or the time commitment for PCR and gel-running. This marker set will facilitate the identification of the functional variations for grain size genes to quickly genotype a large number of cultivars. As shown in the study, a large set of RDP1 accessions was genotyped by these markers to simultaneously compare the effects of different genes, with considerably more capacity than Sanger sequencing (Wang et al. 2015b). In the future, this functional marker set will ultimately facilitate accurate allele mining for both genetic research and breeding applications.

Due to their global origin, the RDP1 accessions represent a wide range of genetic diversity (McCouch et al. 2016), as well as phenotypic diversity for the four grain size traits evaluated in this study, with the trait performance being relatively consistent between the two planting locations. Previously, these same traits were analyzed for the RDP1 grown in Arkansas, USA (Zhao et al. 2011), so we compared our dataset to these results. The correlation coefficients for grain length, grain width, length to width ratio and grain weight were 0.88, 0.89, 0.91 and 0.80, respectively, between the Arkansas and Shanghai data, and 0.72, 0.79, 0.76 and 0.66, respectively, between the Arkansas and Hainan data. Therefore, our data were well correlated with the previous data and expands the germplasm information for public use. The diversity panel can be categorized into the *indica* and *japonica* subspecies based on genotyping with 700,000 SNPs (McCouch et al. 2016). We found that the values of both grain width and grain weight in *japonica* were significantly larger than those of *indica*, suggesting that *japonica* cultivars might contain superior genes/alleles for these two traits. However, the main reason for this difference might not be attributable to the preferential allele distribution of the tested FNPs because the enriched alleles only consistently had a promoting effect for GW2_SNP, GS3_SNP1 and GLW7_InDel. In the future, it will be important to clarify the novel loci for these subspecies differences in segregating populations. Although there is a known close relationship between the *aus* and *indica* subpopulations composing the *indica* subspecies and similarly between *temperate japonica* (*Tej*) and *tropical japonica* (*Trj*) subpopulations composing the *japonica* subspecies, significant difference in grain size were noted between the *Tej* and *Trj* subpopulations, suggesting grain size is an important driver of subpopulation structure (Ali et al. 2011; McClung et al. 2020; Zhao et al. 2011). To investigate this relationship, we used our data to compare the grain size traits of four subpopulations (Table S2 and Table S3), and found significant differences between the *Tej* and *Trj* subpopulations for all four grain size traits but no difference between the *aus* and *indica* subpopulations, consistent with the previous results from different locations. We concluded four FNPs might contribute to the grain size difference between the two *japonica* subpopulations, namely, qLGY3_SNP, GW5_InDel, GLW7_InDel and GL7_InDel1, because the allele ratio is differentiated between the subpopulations (96%, 76%, 86% and 84% for allele 1 in *Tej* and 90%, 83%, 89% and 94% for allele 2 in *Trj* for four FNPs, respectively). This allele distribution might explain why *Tej* accessions have wider but shorter grains in contrast to *Trj* accessions as shown in previous studies (Ali et al. 2011; McClung et al. 2020; Zhao et al. 2011).

Previous GWAS repeatedly identified *GS3* and *GW5* as the major loci for grain size using different collections (Huang et al. 2010; McCouch et al. 2016; Zhao et al. 2011). We also consistently found a significant correlation between grain size traits to the two genes, with a major contribution of *GW5* to grain width and length to width ratio in both two subspecies at both locations. The large contribution of *GW5* was also confirmed in the segregating population, indicating that *GW5* can function as a key target to modulate grain size during breeding. Regarding *GS3*, we found that the C to A mutation was not the only functional allele for grain length because a large effect also was identified for the TG to CC mutation linked to a 3-bp deletion in exon 5. Different deletions in exon 5 of *GS3* were identified to generate short grain rice (Takano-Kai et al. 2013), suggesting the role of the 3-bp deletion in reducing grain length. However, we did not identify a contribution of this allele in the segregating F_2_ population, possibly due to the interaction between the two loci, as discussed below. In addition to the 3-bp deletion, another 1-bp deletion was previously identified in a specific cultivar Chuan 7 (Mao et al. 2010), but we did not find this allele by sequencing 43 accessions, suggesting that it belongs to a rare allele. In the GWAS analysis, *GLW7* could only be identified in the *japonica* cultivars (Si et al. 2016), and we did find a significant contribution of its FNP in the subspecies. In addition, we found a significant contribution of two *GL7* FNPs for nearly all four grain size traits, which was rarely identified by GWAS. This finding suggested that the FNPs might not be linked to SNPs used for GWAS, and our marker set can be used to enhance such analysis in the future.

The performance of some QTLs relied on interactive factors to shape regulatory pathways, and such phenomena have been found for *qLGY3*, *GW6a*, *GLW7*, *GL7*/*GW7* and *GW8* (Liu et al. 2018; Si et al. 2016; Song et al. 2015; Wang et al. 2012). The expression of *GL7*/*GW7* was negatively regulated by *GW8*, and the contribution of *GW8* could only be identified when it was pyramided with the responsive *GW7* allele (Wang et al. 2015a). In our analysis, we found similar situations when *GW5* was combined with several other loci, and the contribution of other loci could only be identified with the responsive *GW5* allele. Therefore, *GW5* might be an important downstream gene coordinating the performance of other loci, and it is important to consider these interactions between these genes when breeding for a particular market class. Such interactions might also affect the performance of other loci during QTL analysis, because we only found an exact colocalization of the QTL with *GW5* but not the other six FNPs tested. Evidence from QTL analysis by Wang et al. (2012) concludes some loci can only be detected in the populations without *GW5* segregation, thus it would be worthwhile to test the contribution of other loci combined with different *GW5* alleles using near isogenic lines in the future.

With the availability of these newly developed functional markers, it is now possible to rationally design the grain size by introgressing or pyramiding different alleles as part of the breeding process. Deciding which genes and alleles to use depends on the desired grain size and allele types of recipient cultivars, since either short, medium or long grain cultivars could be the target depending on the market class. Based on the allele contribution obtained, allele 1 of GW2_SNP, qLGY3_SNP, GS3_SNP1, TGW3_SNP, GW5_InDel, GLW7_InDel, GL7_Dup, and GL7_InDel1 and allele 2 of GS3_SNP2, GS5_InDel, and TGW6_InDel could be used to generate short and bold rice, and their contrasting alleles can be used to generate long and slender rice. To implement MAS, it is first necessary to confirm the allele type absent in the recipient using the marker set developed in this study, and determine which gene(s) to use. The superior alleles of GW2_InDel, GS2_SNP and GL3.1_SNP for large grains can be used for nearly all recipients due to their low frequency. Theoretically, the extent of trait change relies on the number of target alleles used, and a medium grain type could be obtained using some of these target alleles. We listed the allele information of 257 RDP1 accessions for all the tested FNPs (Table S4), thus it will be easier for breeders to select suitable donor accessions based on these FNPs. In China, most of the *japonica* elite cultivars were bred with short and round grains, which could lead to chalkiness, an undesirable grain appearance. Zhao et al. (2018) suggested *japonica* cultivars with a better grain appearance might be bred by introgressing the *gs9* mutation without reducing grain yield and grain yield further improved by pyramiding the *gs3* allele, suggesting higher grain yield and better grain quality could be obtained simultaneously by the proper allele combination. In the future, using the marker set of 14 grain size genes, it will be important to introgress and pyramid the different alleles into the same *japonica* background and explore the best allele combination to design cultivars with a balance between grain yield and grain quality.

## Conclusion

In this study, 21 functional markers were developed for 14 genes regulating grain size and their ability to discriminate different alleles was clarified. A large diversity panel was genotyped with this marker set to determine the allele distribution of the different FNPs. Furthermore, the panel was evaluated for four grain size traits at two planting locations and the contribution of the FNPs to these traits was compared in the *indica* or *japonica* subspecies. Eleven markers were found to be significantly associated with grain size traits, and allele 2 of GW5_InDel could sustain the effects of several other loci. QTL analysis was performed to test the effect of seven polymorphic markers between two cultivars and confirmed the major contribution of *GW5* in determining grain size. This marker set may facilitate both association and linkage analyses for future genetic study, and provides efficient tools for the rational design of grain size as part rice breeding strategies in the future.

## Methods

### Plant Materials

Of the 261 rice cultivars evaluated in this study, 257 were part of the Rice Diversity Panel 1 (RDP1) and obtained from the U.S. Department of Agriculture Genetic Stocks-*Oryza* (Eizenga et al. 2014). The seeds of the RDP1 accession seeds were planted at the Songjiang Experiment Station near Shanghai, China (N 31.03°, E 121.22°) and Lingshui Experiment Station near Hainan, China (N 18.25°, E 109.50°) for trait analysis. Four additional cultivars obtained from China National Rice Research Institute and Zhejiang Academy of Agricultural Sciences were only used to evaluate the marker quality, included *indica* cultivars YYP1 and Baodali and *japonica* cultivars Daohuaxiang and WY3. YYP1 is a new plant type landrace with strong culms and large panicles. Daohuaxiang is a modern elite cultivar, widely planted in China. WY3 and Baodali are cultivars with notably large grains that contain the superior alleles *GW2*/*GL3.1* and *GS2*, respectively. The four cultivars are available from Dr. Lin Zhang upon reasonable request for research purposes. YYP1 was crossed with Nipponbare to generate F_1_ and then selfed to generate F_2_ seeds, and F_2_ seeds were planted at Shanghai for QTL analysis.

### Validation of Allele Variations

Sanger sequencing was performed for some loci to confirm the sequence variation, including the *TGW6* coding region and *GW6a* promoter region in Kasalath, the coding region of *GW2* and *GL3.1* in WY3, and the duplication border of *GL7* in Katy. Using randomly selected RDP1 accessions, four accessions were sequenced in the 5’UTR region of *GLW7* and 43 in the intron 4-exon 5 region of *GS3*. For the remaining allele variations, the sequences were retrieved either from the published data or RiceVarMap2 database (http://ricevarmap.ncpgr.cn/v2/). The candidate functional variations and their flanking sequences were aligned to the reference sequence of Nipponbare (NIP) to locate their positions.

### Development of Allele Specific Markers

For InDel markers, the primers were designed by software Premier primer 5 directly, with a pair of primers spanning the InDel region, and the amplicon size was set to no more than ten times that of the InDel. As the mutation of *qLGY3* led to the loss of the StyI restriction enzyme site, a CAPS marker was designed with primers flanking this mutation. The dCAPS Finder 2.0 tool (http://helix.wustl.edu/dcaps/dcaps.html) was used to design dCAPS markers with permission of one or two mismatches on the primers, and forward primers with inexpensive restriction enzyme sites were selected. To match with the forward primers, Premier Primer 5 was used to select reverse primers. As dCAPS Finder 2.0 cannot recognize small InDels, we inputted the modified SNP-type sequence to facilitate enzyme site finding.

### DNA Extraction, PCR and Gel Electrophoresis

The DNA was extracted by the mini-prep protocol following a previous publication (Xu et al. 2004). A 20 μL system with 2 μL of template DNA, 2 μL of dNTPs (2 mM), 1 μL of each primer (10 μM), 0.3 μL of Taq polymerase, 2 μL of 10 × PCR buffer (Dingguo Biotech, Beijing) and 12.7 μL of distilled water was subjected to PCR for most of the markers. For the GW5_InDel and GS3_SNP1 markers, 10 μL of 2 × PCR premixture (Yisheng Biotech, Shanghai) was used because it amplified better, and supplemented with 2 μL of template DNA, 1 μL of each primer (10 μM) and 6 μL of distilled water. The PCR program was set as follows: an initial denaturation at 94 °C for 3 min, 35 cycles of denaturation at 94 °C for 10 s, annealing at the proper temperature for 20 s, extension at 72 °C for 1 min per kb, and a final extension at 72 °C for 5 min. For dCAPS or CAPS markers, 10 μL of PCR products were used for enzyme analysis with 2 μL of 10 × reaction buffer, 0.3 μL of restriction enzyme (Thermo Fisher Scientific) and 7.7 μL of distilled water to obtain the 20 μL system. After digestion at 37 °C for one hour, 10 μL of product was used for electrophoresis. During gel electrophoresis, 1% agarose gel was used for GW5_InDel and GL7_dup markers, and 3% agarose gel was used for the remaining markers.

### Analysis of Grain Size Related Traits

The seeds of RDP1 accessions and YYP1/NIP F_2_ progeny were harvested at full maturity, and approximately 100 filled grains of each were scattered evenly on a white board equipped with a footlight and imaged with a high-speed scanner (Eloam S500, Shenzhen Eloam Technology Co.,Ltd., Shenzhen P.R.C). The images were analyzed by the SC-G program package (Wanshen, Hangzhou P.R.C), which automatically generated the grain number, grain length, grain width and length to width ratio information. The same set of grains was then weighed, and 1000 grain weight was calculated as follows: (grain weight/grain number) * 1000. The Pearson’s correlation coefficients among traits at two locations were calculated by the R package “corrplot”, and the heatmap was drawn accordingly.

### Trait-Marker Association Analysis

The genotypes of all markers were assigned to each accession of the RDP1 and the allele frequency was calculated accordingly. To facilitate trait comparison, we grouped the RDP1 into *indica* and *japonica* subspecies based on the published population structure (McCouch et al. 2016). The *indica* subspecies included the *admix*-*indica*, *aus* and *indica* subpopulations and the *japonica* subspecies the *admix*-*japonica*, *temperate japonica* and *tropical japonica* subpopulations. Twenty-four accessions were dropped for analysis, including five admixed accessions, eight *aromatic* accessions and eleven accessions without subpopulation assignment. Either the *indica* or *japonica* subspecies was separated into two allele types for each marker, and only markers with more than 15 accessions for minor alleles in one subspecies were chosen to compare the allele effect. Student’s t test was performed by Graphpad Prism 6 software to calculate the significance level of trait differences between alleles, and the trait change rate between alleles was calculated as follows: (allele 2 value – allele 1 value)/allele 1 value. To test the possible interaction between *GW5* and other loci, six markers showing a stable effect on grain width control were selected and each marker will form a four allele combination with the *GW5* marker. The trait value of four allele combinations was subjected to one-way ANOVA, followed by Tukey’s test for multiple comparisons.

### QTL Analysis

One hundred nineteen individuals of the F_2_ population from YYP1 and NIP were genotyped by 123 markers including seven of the newly developed functional markers for grain size (GW2_SNP, GS3_SNP2, GS5_InDel, GW5_InDel, GLW7_InDel, GL7_InDel2 and GW8_InDel), 40 InDels, 62 SSRs and 14 SNPs, which were evenly distributed on the 12 chromosomes. QTL analysis was conducted using QTL IciMapping 4.2 software (Meng et al. 2015), and the genetic distance between markers was calculated by the Kosambi mapping function of the software. The inclusive composite interval mapping of additive (ICIM-ADD) QTL method was used to detect the QTLs. A significant logarithm of the odds (LOD) threshold value was set as 3 for each trait to claim the existence of QTLs. Locations of QTLs were expressed by flanking markers.

## Supplementary information

**Additional file 1: Figure S1.** Alignment of the *GS3* sequence covering the 3-bp deletion and the linked SNPs from 43 randomly selected Rice Diversity Panel 1 accessions. Note that the SNPs are absolutely linked with the 3-bp deletion.

**Additional file 2: Figure S2.** Alignment of the *GLW7* sequence covering two types of deletions from four randomly selected Rice Diversity Panel 1 accessions.

**Additional file 3: Figure S3.** Sketch map illustrating the restriction enzyme sites induced by the forward dCAPS primers. The primer sequences are highlighted in blue, and nucleotide substitutions generating enzyme sites are highlighted in purple. The sequence context of each enzyme site is underlined. Allele 1 stands for the NIP-type sequence, while allele 2 stands for the non-NIP type.

**Additional file 4: Table S1.** Information on 14 grain size genes for marker development.

**Additional file 5: Table S2.** Origin and population structure of the Rice Diversity Panel 1 accessions included in the study based on McCouch et al. (2016).

**Additional file 6: Table S3.** Phenotypic data for the Rice Diversity Panel 1 accessions grown at the Songjiang Experiment Station near Shanghai and the Lingshui Experiment Station near Hainan, China.

**Additional file 7: Table S4.** Allele type of the Rice Diversity Panel 1 accessions genotyped by 20 functional markers.

**Additional file 8: Table S5.** Information on 123 markers used for QTL analysis of the YYP1/NIP F_2_ population.

**Additional file 9: Table S6.** Effects and locations of the QTLs identified from the YYP1/NIP F_2_ population.

## Data Availability

The datasets supporting the conclusions of this article are provided in the article and its additional files. The RDP1 accessions can be ordered from GSOR, and the remaining four rice cultivars are available from Dr. Lin Zhang upon reasonable request.

## Abbreviations

PCR: Polymerase chain reaction
FNPs: Functional nucleotide polymorphisms
QTL: quantitative trait locus
MAS: molecular assisted selection
GWAS: Genome-wide association studies
InDel: Insertion and deletion
SNP: single nucleotide polymorphism
UTR: untranslated region
NIP: Nipponbare
