# Supplementary figures and images for: Grain Size Selection Using Novel Functional Markers Targeting 14 Genes in Rice
Source: Rice (N Y). 2020 Sep 9;13:63. doi: 10.1186/s12284-020-00427-y (PMC7481322; doi:10.1186/s12284-020-00427-y)

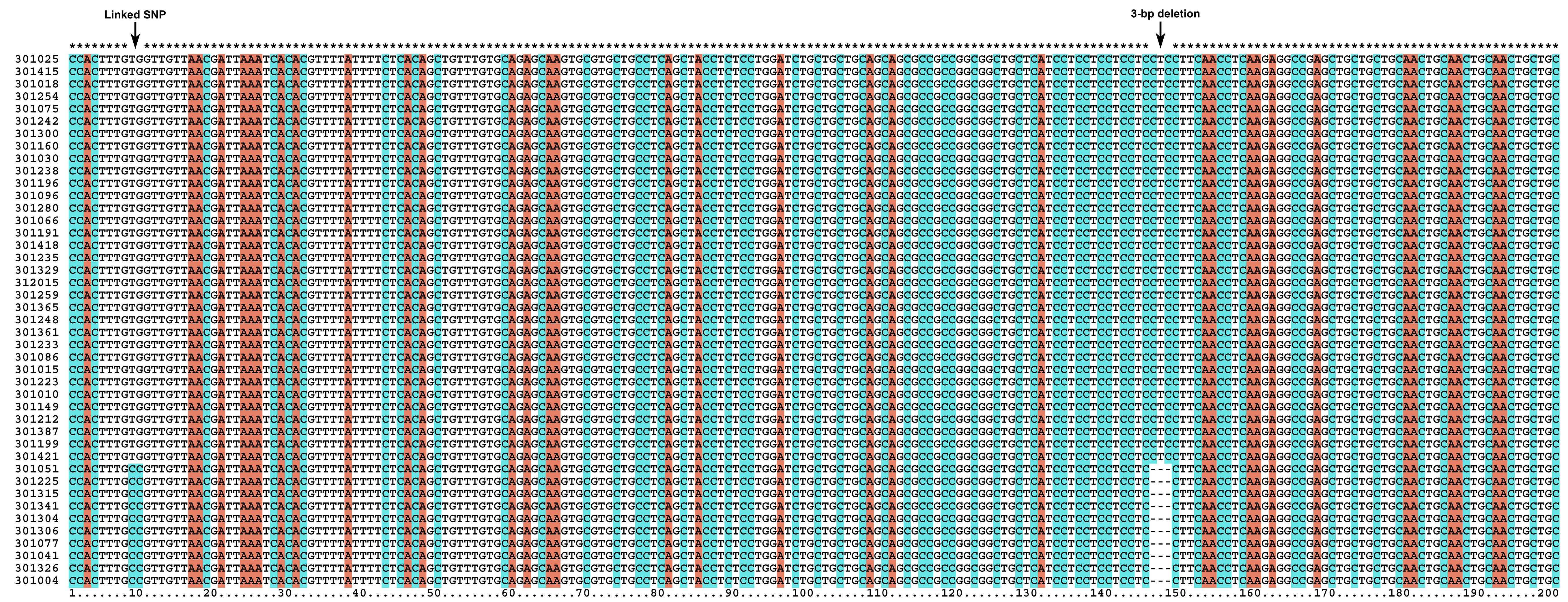

Supplement: Supplementary file 1 — Additional file 1: Figure S1. Alignment of the GS3 sequence covering the 3-bp deletion and the linked SNPs from 43 randomly selected Rice Diversity Panel 1 accessions. Note that the SNPs are absolutely linked with the 3-bp deletion. [file 12284_2020_427_MOESM1_ESM.jpg]

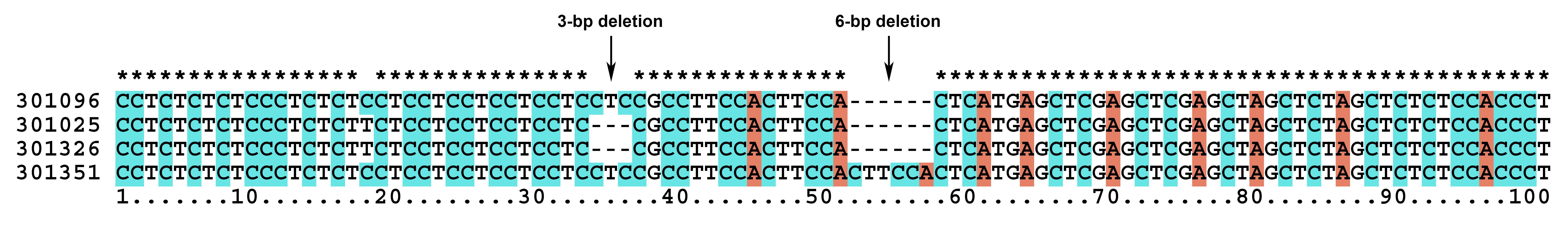

Supplement: Supplementary file 2 — Additional file 2: Figure S2. Alignment of the GLW7 sequence covering two types of deletions from four randomly selected Rice Diversity Panel 1 accessions. [file 12284_2020_427_MOESM2_ESM.jpg]

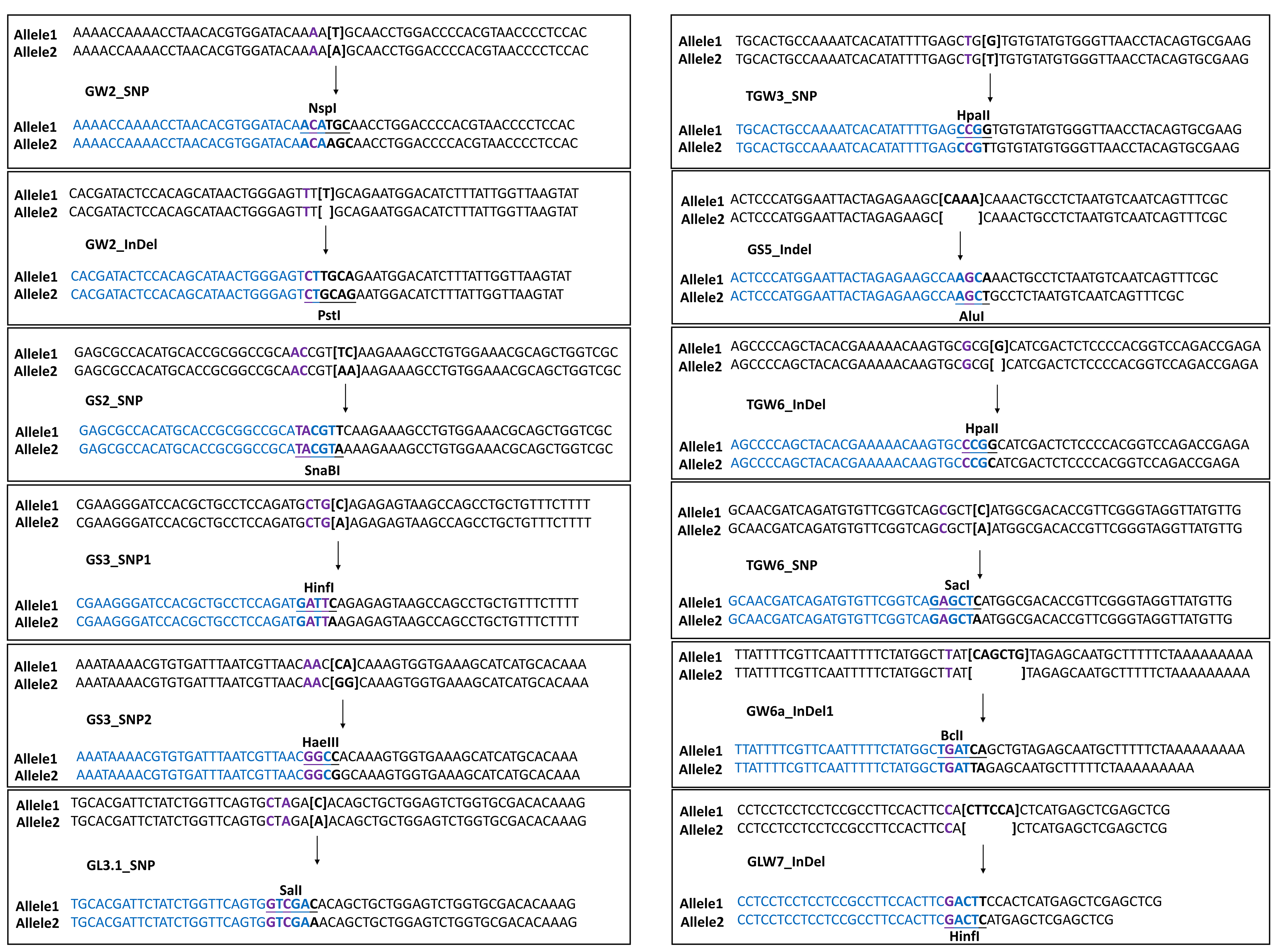

Supplement: Supplementary file 3 — Additional file 3: Figure S3. Sketch map illustrating the restriction enzyme sites induced by the forward dCAPS primers. The primer sequences are highlighted in blue, and nucleotide substitutions generating enzyme sites are highlighted in purple. The sequence context of each enzyme site is underlined. Allele 1 stands for the NIP-type sequence, while allele 2 stands for the non-NIP type. [file 12284_2020_427_MOESM3_ESM.jpg]
